# Predictors of Psychological Stress and Infertility Among Reproductive‐Age Women in Jammu and Kashmir: A Cross‐Sectional Study

**DOI:** 10.1002/hsr2.73482

**Published:** 2026-11-05

**Authors:** Kritika Sharma, Indu Bhushan, Srinivas Sistla

**Affiliations:** ^1^ School of Biotechnology Shri Mata Vaishno Devi University Katra Jammu and Kashmir India; ^2^ Division of Research and Innovation University of Arkansas Fayetteville Arkansas USA

**Keywords:** infertility, psychological stress, reproductive tract infections

## Abstract

**Background and Aims:**

Infertility affects nearly one in six individuals globally, with a substantial proportion of cases remaining idiopathic. Emerging evidence suggests that psychological stress may influence reproductive outcomes through neuroendocrine and immune pathways, potentially influencing the vaginal microbiome and the reproductive microenvironment. This study aimed to assess the prevalence of stress and infertility and examine their association among women of reproductive age in Jammu and Kashmir.

**Methods:**

A cross‐sectional study was conducted among 1068 married women aged 18–48 years in Jammu and Kashmir, including women with idiopathic infertility and fertile controls. Data on sociodemographic characteristics, perceived stress, symptoms of reproductive tract infections (RTIs), sleep patterns, and physical stress manifestations were collected using a structured interviewer‐administered questionnaire. Binary logistic regression analysis was performed to identify factors associated with psychological stress and infertility.

**Results:**

Overall, 37.6% of participants reported stress, while 29.3% had infertility. Stress was significantly associated with older age groups, employment status, lower financial status, RTI symptoms, physical stress manifestations, sleep duration, and infertility. Women with infertility had markedly higher odds of stress. Infertility was significantly associated with a history of stress, age, employment status, financial status, RTI symptoms, and sleep duration.

**Conclusion:**

Psychological stress is strongly associated with infertility and related health and socioeconomic factors among women in this population. These findings highlight the potential interplay between psychological, reproductive, and social factors in reproductive health. Further longitudinal studies are needed to clarify causal pathways and inform targeted interventions.

**Abbreviations**
AOR: adjusted odds ratio
CI: confidence interval
PSS: perceived stress scale
RTIs: reproductive tract infections

## 1 Introduction

Infertility is a major global reproductive health concern, affecting approximately one in six individuals during their reproductive years, as reported by the World Health Organization (WHO) [1]. Beyond its medical implications, it imposes substantial emotional, psychological, and socio‐economic burdens on affected individuals and couples. Clinically, infertility is defined as the inability to achieve pregnancy after 12 months of regular, unprotected sexual intercourse [2]. Female infertility arises from diverse etiologies, including genetic abnormalities, hormonal and endocrine dysfunction, anatomical defects, infections, environmental exposures, and lifestyle factors such as obesity, smoking, and alcohol consumption, all of which may impair reproductive function [3, 4, 5]. However, in nearly 30% of cases, routine clinical evaluation fails to identify a definitive cause, leading to a diagnosis of idiopathic infertility, highlighting important gaps in current understanding of reproductive failure [6, 7].

Growing evidence suggests that subtle biological and psychosocial factors, including immune dysregulation, impaired endometrial receptivity, epigenetic modifications, and alterations in microbial ecosystems, may contribute to unexplained infertility [8, 9]. In particular, the vaginal microbiome plays a crucial role in maintaining reproductive tract health. A Lactobacillus‐dominant environment preserves an acidic pH and protects against pathogenic colonization through the production of lactic acid and antimicrobial compounds. Disruptions in this microbial balance have been associated with adverse reproductive outcomes in previous studies [10].

Psychological stress has emerged as an important modulator of reproductive health and may influence vaginal microbial stability through neuroendocrine and immune pathways [11, 12]. Chronic stress activates the hypothalamic–pituitary–adrenal (HPA) axis, leading to sustained cortisol secretion, which can alter mucosal immunity and epithelial integrity. These changes may potentially disrupt vaginal microbial balance by reducing protective *Lactobacillus* species and promoting opportunistic organisms, thereby increasing susceptibility to reproductive tract infections [10, 13, 14]. Additionally, stress‐related alterations in gut microbiota and estrogen metabolism may further contribute to microbial imbalance [15, 16, 17]. The proposed pathways linking stress, microbial alterations, and reproductive health are summarized in Figure 1.

**Figure 1:**
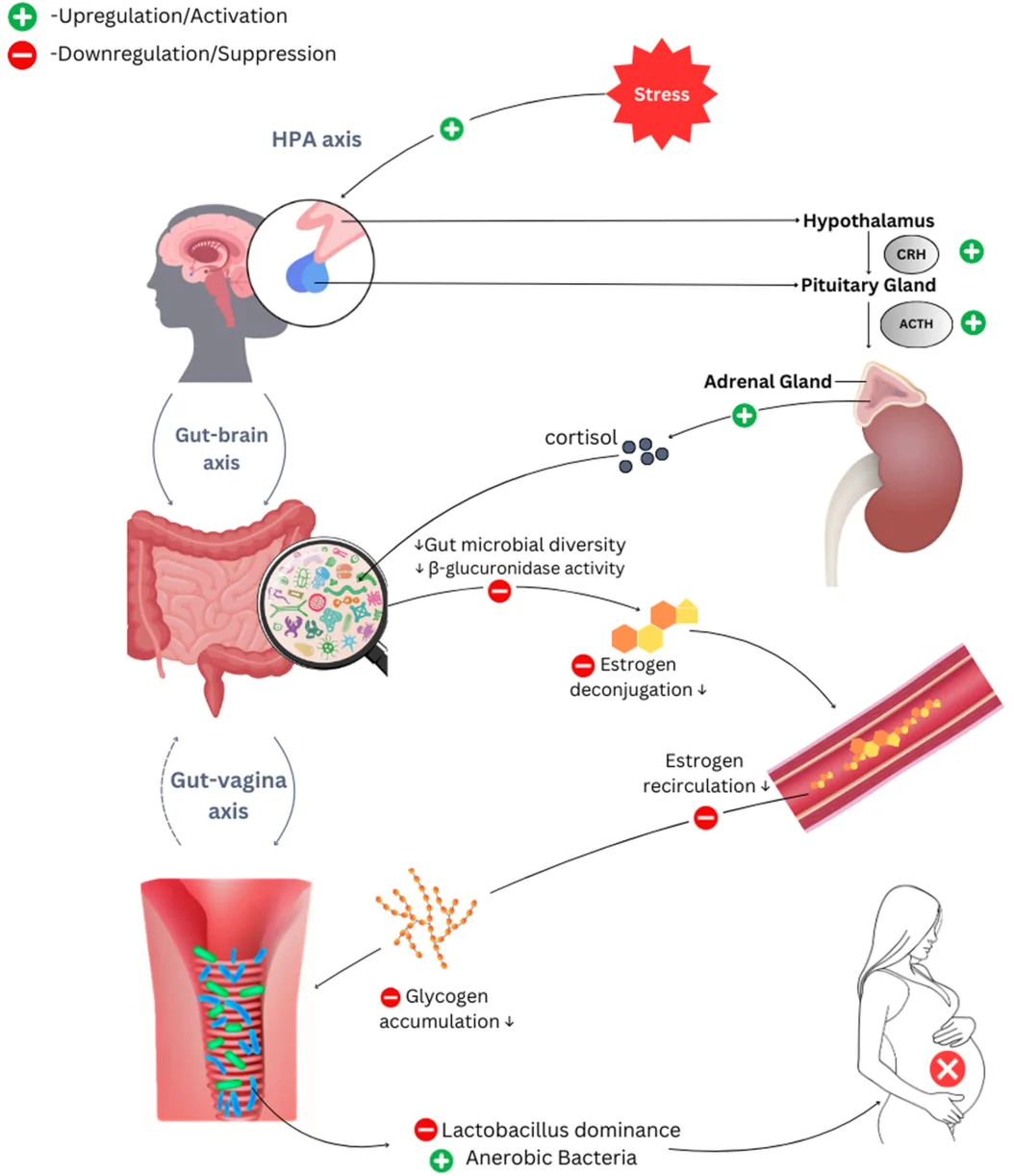
Mechanistic overview of stress‐induced alterations in the gut‐brain and gut‐vaginal axes affecting vaginal microbiome composition and reproductive health. The figure illustrates the proposed pathway through which psychological stress may influence neuroendocrine and immune responses, gut microbiota, and the vaginal microbiome, with potential consequences for reproductive health. Arrows indicate the proposed direction of these interactions. HPA, hypothalamic–pituitary–adrenal.

In addition to biological pathways, psychological stress is closely intertwined with social disadvantage, sleep deprivation, occupational strain, and reproductive health symptoms, all of which may independently or synergistically influence fertility outcomes [18, 19, 20, 21]. Jammu and Kashmir represents a unique sociocultural and geographic setting where prolonged exposure to conflict, socioeconomic challenges, and healthcare disparities may contribute to increased psychological stress and mental health burden [22, 23]. Studies from the region have reported a high prevalence of psychological distress, including anxiety, depression, and post‐traumatic stress symptoms, reflecting sustained psychosocial stressors in the population [24]. Although the vaginal microbiome is discussed here as a potential biological pathway linking psychological stress with reproductive health and infertility, microbiome composition was not directly assessed in the present study. Instead, symptoms suggestive of reproductive tract infections (RTIs), obtained through the questionnaire, were considered as an indirect indicator of reproductive tract disturbances that may be associated with microbial dysbiosis. Despite this, region‐specific evidence examining the relationship between psychological stress and infertility remains limited.

### 1.1 Research Questions

• What is the prevalence of psychological stress among reproductive‐age women in Jammu and Kashmir?
• What sociodemographic, reproductive, and health‐related factors are associated with psychological stress in this population?
• What is the prevalence of idiopathic infertility, and which factors are associated with infertility?
• Is psychological stress associated with idiopathic infertility after adjustment for relevant covariates?

Therefore, to address these questions, the present study aimed to estimate the prevalence of psychological stress and evaluate its association with idiopathic infertility among women in Jammu and Kashmir.

## 2 Materials and Methods

### 2.1 Ethical Clearance

Ethical approval for this study was granted by the Institutional Ethics Review Board (IERB) of Shri Mata Vaishno Devi University (IERB serial no. SMVDU/IERB/2024/09/01). Informed consent was obtained from all participants before their inclusion in the study.

### 2.2 Source Population, Inclusion, and Exclusion Criteria

This hospital‐based, multicentric cross‐sectional study was conducted over 8 months (April 2025–December 2025) in the outpatient departments of Obstetrics and Gynaecology at Government Medical College Jammu, Shri Mata Vaishno Devi Narayana Superspeciality Hospital Katra, and Government Sub‐District Hospital Katra. A total of 1068 women were included in the study, comprising 313 women with idiopathic infertility and 755 fertile controls. Participants were recruited using a convenience sampling approach during the study period. Women of reproductive age (18–48 years) who were married, sexually active, and provided written informed consent were eligible for participation. For inclusion in the infertility group, women were required to report regular, unprotected sexual intercourse for at least 12 months without achieving pregnancy and to have been identified as having idiopathic infertility during their routine clinical evaluation. Women who had conceived and were attending the participating hospitals for antenatal care were included as fertile controls.

Women who were postmenopausal or had known comorbid conditions such as thyroid disorders, diabetes, or other chronic illnesses were excluded from the study. Women who did not meet the specified eligibility criteria or who did not provide written informed consent were also excluded.

The sample size was calculated by the following formula [25].

$$n=\frac{{{(Z}_{\frac{\propto }{2}})}^{2}*p(1-p)}{d^{2}}$$

Where, *Z*_*α/2*_ is the standard normal variable value for a 95% confidence interval (CI), with *α* = 0.05, so *Z*_*α/2*_ = 1.96; *p* is the estimated prevalence of stress, set at 50% as a conservative estimate, and *d* is the margin of error (5%).

The minimum calculated sample size was 384 participants. Recruitment was continued beyond the minimum to increase the number of eligible participants available for analysis and to account for exclusions and non‐response during the study period. Of the 1200 women approached, 1175 provided informed consent, and after exclusion of 107 participants based on the predefined criteria, 1068 participants were included in the final analysis.

### 2.3 Outcome Measurement

The primary outcome variable of the study was psychological stress and infertility status. Psychological stress was assessed using a structured questionnaire adapted from the Perceived Stress Scale (PSS) [26]. Stress perception over the preceding month was evaluated using four items reflecting feelings of unpredictability, lack of control, and overload. Participants responded to each item on a four‐point Likert scale ranging from never (0) to very often [3]. Positively worded items were reverse‐scored, while negatively worded items were scored directly. Individual item scores were summed to generate a composite perceived stress score ranging from 0 to 12. Based on established cut‐offs, stress levels were categorized as low (0–4), moderate [5, 6, 7, 8], and high [9, 10, 11, 12].

For regression analysis, stress was further classified as a binary variable, with participants scoring in the moderate or high categories considered to have stress, and those with low scores considered non‐stressed. In addition to perceived stress scores, supportive indicators including self‐reported stress, physical symptoms (such as headache, fatigue, blood pressure fluctuations, and digestive issues), and sleep duration were collected to contextualize stress exposure [27].

### 2.4 Study Instrument and Data Collection

A semi‐structured, interviewer‐administered questionnaire was developed in English, Hindi, and Urdu to accommodate linguistic diversity among participants. Trained researchers fluent in these languages facilitated data collection, ensuring participants fully understood the questionnaire. A pilot study was conducted on 10% of the sample size to refine the questionnaire based on participant feedback. Information on sociodemographic factors, perceived stress levels, symptoms of reproductive tract infection (RTI), and infertility status was collected.

Participants reporting symptoms suggestive of RTIs were recorded based on self‐reported information obtained through the questionnaire. These variables were included as indicative clinical symptoms rather than laboratory‐confirmed diagnoses. If one or more of the following symptoms appeared, RTI was assumed to be present: unusual vaginal discharge, genital itching and burning, burning micturition, Lower Abdominal Pain, excessive bleeding, genital ulcers, and Lower Back Pain [28]. The quality of compiled data was ensured through on‐site supervision, spot‐checks, and back‐checks.

### 2.5 Data Analysis

The data were coded, entered into Microsoft Excel (ver. 2024), and exported to SPSS (version 27.0) for analysis. Descriptive statistics were used to evaluate the background characteristics of the participants. Binary logistic regression was used to assess the association between sociodemographic, health, and lifestyle factors and the outcomes of infertility and stress, respectively. Adjusted odds ratios (AORs) with 95% confidence intervals (CIs) were reported. For the stress model, the outcome was coded as 0 = no stress and 1 = stress; for the infertility model, it was coded as 0 = fertile and 1 = infertile. Categorical variables were entered into the models using defined reference categories. All statistical tests were two‐sided, with statistical significance set at *p* < 0.05.

## 3 Results

### 3.1 Participant Characteristics

A total of 1200 women were approached during the study period, of whom 1175 consented to participate. Following exclusion of 107 participants based on the predefined eligibility criteria, 1068 women were included in the final analysis. Of these, 313 (29.3%) had idiopathic infertility, including 235 (75.1%) with primary infertility and 78 (24.9%) with secondary infertility, while 755 (70.7%) were fertile controls.

The mean age of the participants was 28.89 ± 6.06 years. Of the total participants, 554 (51.9%) resided in urban areas and 514 (48.1%) in rural areas. Most participants had completed at least higher secondary education, and 483 (45.2%) were employed, while 585 (54.8%) were unemployed. The majority of participants were Hindu (693, 64.9%), followed by Sikh (191, 17.9%) and Muslim (174, 16.3%) participants, while Christians constituted a small proportion (10, 0.9%). Approximately half of the participants belonged to the Below Poverty Line (BPL) category (544, 50.9%), while 524 (49.1%) were classified as Above Poverty Line (APL).

Regarding health‐related characteristics, 484 (45.3%) participants reported symptoms suggestive of reproductive tract infections (RTIs), while 584 (54.7%) did not. Stress‐related physical symptoms were reported by 328 (30.7%) participants, whereas 740 (69.3%) did not report such symptoms. Most participants (615, 57.6%) reported sleeping for 6–8 h per night.

### 3.2 Prevalence and Characteristics of Psychological Stress

Overall, 402 of 1068 participants (37.6%) were classified as having psychological stress, while 666 (62.4%) were not. The prevalence of stress varied across demographic and health‐related characteristics. Stress was reported by 22.6% of women aged 18–20 years, 38.0% of those aged 21–30 years, 46.7% of those aged 31–40 years, and 15.2% of those aged 41–48 years. Higher proportions of stress were also observed among women reporting RTI symptoms (57.2%) and physical stress symptoms (78.7%) compared with those without these symptoms (21.4% and 19.5%, respectively). Stress was reported by 45.6% of women belonging to the BPL category compared with 29.4% among those in the APL category. Detailed distributions of stress across the study characteristics are presented in Table 2.

### 3.3 Factors Associated With Psychological Stress

A binary logistic regression analysis was performed to examine factors associated with perceived stress among the participants. The model demonstrated an acceptable overall fit (−2 Log Likelihood = 815.136). The Cox and Snell *R* ^2^ (0.430) and Nagelkerke *R* ^2^ (0.585) values indicate that the variables included in the model accounted for approximately 43%–59% of the variation in perceived stress. The summary statistics for the regression model are presented in Table 1.

**Table 1:** Logistic regression model summary showing model fit for predictors of stress.

Model summary			
Step	−2 Log likelihood	Cox and Snell *R* square	Nagelkerke *R* square
1*	815.136	0.430	0.585

* Step 1 refers to the first step of the binary logistic regression model, which includes all predictor variables entered simultaneously to assess their combined contribution to predicting stress.

After adjustment for the variables included in the model, age, educational attainment, religion, employment status, financial status, RTI symptoms, physical stress symptoms, and sleep duration showed significant associations with psychological stress. Women aged 31–40 years (AOR: 3.425; 95% CI: 1.719–6.825; *p *< 0.001) and 41–48 years (AOR: 4.906; 95% CI: 2.384–10.095; *p *< 0.001) had higher odds of stress compared with those aged 18–20 years. Primary education (AOR: 0.381; 95% CI: 0.149–0.976; *p *= 0.04) and graduation (AOR: 0.540; 95% CI: 0.309–0.943; *p *= 0.03) were associated with lower odds of stress compared with no formal schooling.

Muslim (AOR: 0.104; 95% CI: 0.021–0.512; *p *= 0.005) and Sikh women (AOR: 0.121; 95% CI: 0.023–0.623; *p *= 0.01) had lower odds of stress compared with Hindu women. Employment status was also associated with stress; employed women had higher odds of stress than unemployed women (AOR: 1.72; 95% CI: 1.12–2.26; *p *= 0.002). Women belonging to the BPL category had higher odds of stress than those in the APL category (AOR: 2.93; 95% CI: 1.93–4.46; *p *< 0.001).

Health‐related factors showed strong associations with stress. Women reporting symptoms suggestive of RTIs had higher odds of stress (AOR: 2.800; 95% CI: 1.937–4.049; *p *< 0.001), while those reporting physical stress symptoms had substantially higher odds (AOR: 8.839; 95% CI: 5.967–13.092; *p *< 0.001. Sleep duration showed a significant association, with women sleeping < 4 h (AOR: 4.85; 95% CI: 1.82–12.91; *p* = 0.002) and 4–6 h (AOR: 5.07; 95% CI: 2.73–9.44; *p *< 0.001) having higher odds compared to those sleeping ≥ 8 h, while no significant difference was observed for 6–8 h (AOR: 1.25; 95% CI: 0.73–2.14; *p* = 0.46). Detailed results of the multivariable regression analysis are presented in Table 2.

**Table 2:** Influence of selected sociodemographic and health factors on stress among women of reproductive age in Jammu and Kashmir.

Variables		Symptoms of stress		AOR	95% CI	*p* value
	Total	Yes *n* (%) (*N* = 402)	No *n* (%) (*N* = 666)			
Age (in years)						
18–20	53	12 (22.6)	41 (77.4)	1 (Reference)		
21–30	722	274 (38.0)	448 (62.0)	1.639	(0.646–4.158)	0.30
31–40	227	106 (46.7)	121 (53.3)	3.425	(1.719–6.825)	**< 0.001**
41–48	66	10 (15.2)	56 (84.8)	4.906	(2.384–10.095)	**< 0.001**
Residence						
Rural	514	179 (34.8)	335 (65.2)	1 (Reference)		
Urban	554	223 (40.3)	331 (59.7)	0.991	(0.689–1.426)	0.96
Educational qualification						
No schooling	54	20 (37.0)	34 (63.0)	1 (Reference)		
Primary education	100	35 (35.0)	65 (65.0)	0.381	(0.149–0.976)	**0.04**
High school	193	54 (28.0)	139 (72.0)	0.487	(0.230–1.032)	0.06
Higher secondary	309	112 (36.2)	197 (63.8)	0.595	(0.317–1.118)	0.11
Graduation	254	105 (41.3)	149 (58.7)	0.540	(0.309–0.943)	**0.03**
Postgraduate	158	76 (48.1)	82 (51.9)	1.090	(0.620–1.913)	0.77
Religion						
Hindu	693	234 (33.8)	459 (66.2)	1 (Reference)		
Muslim	174	88 (50.6)	86 (49.4)	0.104	(0.021–0.512)	**0.005**
Sikh	191	74 (38.7)	117 (61.3)	0.121	(0.023–0.623)	**0.01**
Christian	10	6 (60.0)	4 (40.0)	0.596	(0.117–3.034)	0.53
Employment status						
Unemployed	585	178 (30.4)	407 (69.6)	1 (Reference)		
Employed	483	224 (46.4)	259 (53.6)	1.72	(1.12–2.26)	**0.002**
Financial status						
APL	524	154 (29.4)	370 (70.6)	1 (Reference)		
BPL	544	248 (45.6)	296 (54.4)	2.93	(1.93–4.46)	**< 0.001**
Symptoms of RTI						
No	584	125 (21.4)	459 (78.6)	1 (Reference)		
Yes	484	277 (57.2)	207 (42.8)	2.800	(1.937–4.049)	**< 0.001**
Physical stress manifestations						
No	740	144 (19.5)	596 (80.5)	1 (Reference)		
Yes	328	258 (78.7)	70 (21.3)	8.839	(5.967–13.092)	**< 0.001**
Hours of sleep						
8 Or more hours	144	23 (16.0)	121 (84.0)	1 (Reference)		
6–8 h	615	135 (22.0)	480 (78.0)	1.245	0.726–2.139	0.43
4–6 h	246	189 (76.8)	57 (23.2)	5.072	2.725–9.441	**< 0.001**
Less than 4 h	63	55 (87.3)	8 (12.7)	4.845	1.818–12.911	**0.002**
Infertility history						
No	755	177 (23.4)	578 (76.6)	1 (Reference)		
Yes	313	225 (71.9)	88 (28.1)	8.425	(5.241–13.544)	**< 0.001**

*Note:* The bold values indicate the statistical significance of *p* < 0.05.

### 3.4 Prevalence and Factors Associated With Idiopathic Infertility

Of the 1068 participants, 313 (29.3%) had idiopathic infertility, including 235 (22.0%) with primary infertility and 78 (7.3%) with secondary infertility, while 755 (70.7%) were fertile controls.

A second binary logistic regression model was fitted with infertility status as the dependent variable. The model demonstrated an acceptable overall fit (−2 Log Likelihood = 724.233), with Cox and Snell *R* ^2^ of 0.412 and Nagelkerke *R* ^2^ of 0.588, indicating that the included variables accounted for approximately 41%–59% of the variation in infertility outcomes. The detailed results of the regression analysis are presented in Table 3.

**Table 3:** Logistic regression model summary showing model fit for predictors of infertility.

Model summary			
Step	−2 Log likelihood	Cox and Snell *R* square	Nagelkerke *R* square
1*	724.233	0.412	0.588

* Step 1 refers to the first step of the binary logistic regression model, which includes all predictor variables entered simultaneously to assess their combined contribution to predicting infertility.

After adjustment, age, employment status, financial status, RTI symptoms, physical stress manifestations, stress history, and selected categories of education, religion, and sleep duration were associated with infertility. Women aged 21–30 years (AOR: 2.560; 95% CI: 1.235–5.436; *p *= 0.01), 31–40 years (AOR: 2.797; 95% CI: 1.845–7.988; *p *= 0.006), and 41–48 years (AOR: 1.316; 95% CI: 1.016–6.674; *p *= 0.57) had higher odds of infertility than those aged 18–20 years. Among education and religion categories, higher odds were observed among women with high school education (AOR: 2.161; 95% CI: 1.042–4.480; *p *= 0.04), while Christian women had lower odds compared with Hindu women (AOR: 0.093; 95% CI: 0.023–0.383; *p *< 0.001). Employed women had higher odds of infertility than unemployed women (AOR: 5.424; 95% CI: 3.373–8.724; *p *< 0.001), while women belonging to the BPL category also had higher odds of infertility (AOR: 2.674; 95% CI: 1.729–4.136; *p *< 0.001). Women reporting symptoms suggestive of RTIs had higher odds of infertility (AOR: 4.364; 95% CI: 2.941–6.475; *p *< 0.001), as did women reporting physical stress manifestations (AOR: 3.328; 95% CI: 1.982–5.500; *p *< 0.001). Sleep duration was also associated with infertility, with women sleeping 4–6 h having higher odds of infertility than those sleeping less than 4 h (AOR: 2.688; 95% CI: 1.751–4.132; *p *< 0.001), while those sleeping 6–8 h (AOR: 0.756; 95% CI: 0.583–0.994; *p *= 0.04) and 8 or more hours (AOR: 0.445; 95% CI: 0.278–0.744; *p *= 0.003) had lower odds. Table 4 details the association between sociodemographic, health factors, and infertility among the participants.

**Table 4:** Influence of selected sociodemographic and health factors on infertility among women of reproductive age in Jammu and Kashmir.

Variables		Status of infertility		AOR	95% CI	*p* value
	Total	Yes *n* (%) (*N* = 313)	No *n* (%) (*N* = 755)			
Age (in years)						
18–20	53	2 (3.8)	51 (96.2)	1 (Reference)		
21–30	722	225 (31.2)	497 (68.8)	2.560	(1.235–5.436)	**0.01**
31–40	227	74 (32.6)	153 (67.4)	2.797	(1.845–7.988)	**0.006**
41–48	66	12 (18.2)	54 (81.8)	1.316	(1.016–6.674)	0.57
Residence						
Rural	514	138 (26.8)	376 (73.2)	1 (Reference)		
Urban	554	175 (31.6)	379 (68.4)	0.978	(0.666–1.437)	0.91
Educational qualification						
No Schooling	54	15 (27.8)	39 (72.2)	1 (Reference)		
Primary education	100	40 (40.0)	60 (60.0)	0.912	(0.358–2.323)	0.85
High school	193	32 (16.6)	161 (83.4)	2.161	(1.042–4.480)	**0.04**
Higher secondary	309	103 (33.3)	206 (66.7)	0.516	(0.253–1.053)	0.07
Graduation	254	69 (27.2)	185 (72.8)	1.431	(0.785–2.609)	0.24
Postgraduate	158	54 (34.2)	104 (65.8)	1.089	(0.590–2.010)	0.78
Religion						
Hindu	693	207 (29.9)	486 (70.1)	1 (Reference)		
Muslim	174	85 (48.9)	89 (51.1)	0.104	(0.044–1.276)	0.09
Sikh	191	16 (8.4)	175 (91.6)	0.121	(0.085–2.670)	0.40
Christian	10	5 (50.0)	5 (50.0)	0.093	(0.023–0.383)	**< 0.001**
Employment status						
Unemployed	585	200 (34.2)	385 (65.8)	1 (Reference)		
Employed	483	113 (23.4)	370 (76.6)	5.424	(3.373–8.724)	**< 0.001**
Financial status						
APL	524	187 (35.7)	337 (64.3)	1 (Reference)		
BPL	544	126 (23.2)	418 (76.8)	2.674	(1.729–4.136)	**< 0.001**
Symptoms of RTI						
No	584	67 (11.5)	517 (88.5)	1 (Reference)		
Yes	484	246 (50.8)	238 (49.2)	4.364	(2.941–6.475)	**< 0.001**
Physical stress symptoms						
No	740	124 (16.8)	616 (83.2)	1 (Reference)		
Yes	328	189 (57.6)	139 (42.4)	3.328	(1.982–5.5)	**< 0.001**
Hours of sleep						
Less than 4 h	63	38 (60.3)	25 (39.7)	1 (Reference)		
4–6 h	246	132 (53.7)	114 (46.3)	2.688	(1.751–4.132)	**< 0.001**
6–8 h	615	127 (20.7)	488 (79.3)	0.756	(0.583–0.994)	**0.04**
8 Or more hours	144	26 (18.1)	118 (81.9)	0.445	(0.278–0.744)	**0.003**
Perceived stress						
No	666	88 (13.2)	578 (86.8)	1 (Reference)		
Yes	402	225 (56.0)	177 (44.0)	9.129	(2.29–36.56)	**< 0.001**

*Note:* The bold values indicate the statistical significance of *p* < 0.05.

### 3.5 Association Between Psychological Stress and Idiopathic Infertility

Psychological stress was strongly associated with idiopathic infertility in the study population. In the model with stress as the outcome, women with infertility had markedly greater odds of reporting stress than those without infertility (AOR: 8.425; 95% CI: 5.241–13.544; *p* < 0.001). Similarly, when infertility was considered as the outcome, a history of stress was associated with increased odds of idiopathic infertility (AOR: 9.129; 95% CI: 2.29–36.56; *p* < 0.001).

Taken together, these findings suggest a possible bidirectional relationship between psychological stress and idiopathic infertility, in which stress may be associated with infertility while infertility itself may contribute to psychological stress. However, the cross‐sectional design prevents establishing the temporal direction or causality of this relationship.

## 4 Discussion

The present study examined the factors contributing to psychological stress and infertility and their association with each other among women of reproductive age in the Union Territory of Jammu and Kashmir. Psychological stress was reported by 37.6% of participants, while 29.3% had infertility. The findings indicate a strong relationship between psychological stress and infertility. Women with infertility were more likely to report psychological stress, while a history of stress was also strongly associated with infertility. Rather than being explained by a single factor, these findings point to an interplay of reproductive, socioeconomic, and health‐related factors that may shape both psychological well‐being and reproductive health in this population.

Age was associated with psychological stress, with women in the older age groups showing higher odds of stress compared with the youngest age group. This finding is consistent with that of Osmanovic–Thunstrom et al. [29], who reported increasing levels of perceived stress with increasing age among older adults. This may reflect the accumulation of health, family, social, and economic pressures with increasing age, which may contribute to psychological distress [29, 30]. Age was also associated with infertility, consistent with the established association between advancing age and declining reproductive potential reported by Kamath and Bhattacharya [31] and te Velde and Pearson [32], largely related to reductions in ovarian reserve and oocyte quality. Thus, age appears to be relevant to both psychological and reproductive outcomes, although the underlying pathways are likely to be different.

Among the health‐related factors, RTI symptoms showed a strong association with both psychological stress and infertility. Women reporting symptoms suggestive of RTIs had higher odds of psychological stress (AOR: 2.800; 95% CI: 1.937–4.049; *p *< 0.001). This relationship may be bidirectional, as RTIs may contribute to physical discomfort and emotional distress, while chronic stress can impair immune function and potentially increase susceptibility to infections. Previous studies have similarly reported associations between reproductive tract infections, vulvovaginal infections, and psychological distress [21, 30, 33]. Similarly, women with RTI symptoms had substantially higher odds of infertility (AOR: 4.364; 95% CI: 2.941–6.475; *p *< 0.001), in line with previous studies demonstrating that vaginal and genital tract infections, including bacterial vaginosis and candidiasis, are associated with delayed conception and adverse reproductive outcomes [34, 35, 36]. Such infections may impair fertility through inflammatory damage to reproductive tissues, disruption of the vaginal microenvironment, and reduced endometrial receptivity. Physical manifestations of stress, including complaints such as headaches, fatigue, and blood pressure fluctuations, were strongly associated with psychological stress (AOR: 8.84; 95% CI: 5.967–13.092; *p *< 0.001). reflecting somatic manifestations of psychological distress. Prolonged stress is known to produce physical symptoms through dysregulation of neuroendocrine and cardiovascular pathways [36, 37, 38]. Physical manifestations of stress were also significantly associated with infertility (AOR: 3.328; 95% CI: 1.982–5.500; *p *< 0.001), These symptoms may serve as clinical indicators of underlying psychological distress and further underscore the multifaceted link between stress and reproductive health.

The relationship between stress and infertility was one of the most important findings of the study. Women with a history of stress had substantially higher odds of infertility, while women with infertility also had markedly higher odds of psychological stress. This pattern is consistent with previous evidence identifying infertility as a significant psychosocial burden, with Patel et al. [37, 38, 39, 40] reporting substantial stress among women and couples experiencing infertility. Stress has been proposed to affect reproductive function through neuroendocrine and immune pathways, including alterations in hypothalamic–pituitary–gonadal axis activity and cortisol regulation [41]. At the same time, the experience of infertility itself can generate persistent emotional distress, particularly in sociocultural settings where childbearing is closely linked with family expectations and social identity. The strong associations observed in both models therefore suggest a potentially bidirectional relationship between stress and infertility, although causality cannot be established from the present study.

Sleep duration was associated with both psychological stress and infertility in the present study, although the pattern did not show a consistent dose–response relationship across the sleep categories. In the stress model, women sleeping for less than 8 h had higher odds of experiencing stress compared with those sleeping for 8 or more hours, with the strongest associations observed among women sleeping for 4–6 h and less than 4 h. A similar relationship between shorter sleep duration and psychological stress has been reported by Hirotsu et al. [42], who described a reciprocal relationship between sleep disturbance and stress, with psychological stress affecting sleep and inadequate sleep potentially contributing to stress‐related physiological and emotional changes. Similarly, in the infertility model, women sleeping for 4–6 h had higher odds of infertility, whereas those sleeping 6–8 h and 8 or more hours had lower odds compared with women sleeping less than 4 h. This finding is partly consistent with the findings of Caetano et al. and Li et al. [43, 44], which reported an association between sleep disturbances and female infertility. However, the non‐linear pattern observed in the present study differs from a simple assumption that progressively shorter sleep is associated with progressively greater infertility risk. This pattern may have been observed due to the relatively small number of women in less‐than‐4‐h category kept as the reference.

Socioeconomic circumstances provided another important context for both stress and infertility. Women belonging to the BPL category had higher odds of psychological stress as well as infertility compared with women in the APL category. Financial insecurity can contribute directly to psychological strain and may also influence healthcare‐seeking behavior, access to reproductive services, and the ability to meet treatment‐related expenses. Previous research has similarly highlighted the relationship between socioeconomic disadvantage, mental health, and access to fertility care [18, 45, 46]. These findings are particularly relevant in the context of Jammu and Kashmir, where socioeconomic circumstances and access to healthcare may vary across populations [47]. Employment status was also associated with both psychological stress and infertility, although the association was considerably stronger for infertility. Employed women had higher odds of psychological stress than unemployed women (AOR: 1.72; 95% CI: 1.12–2.26; *p *= 0.002), while the odds of infertility were substantially higher among employed women (AOR: 5.424; 95% CI: 3.373–8.724; *p *< 0.001). The relationship between employment and psychological well‐being and reproductive health may be influenced by differences in workload, time constraints, job demands, work schedules, and the ability to balance occupational and family responsibilities. Evidence from previous studies suggests that adverse psychosocial conditions at work, including high job demands and low job control, may be associated with reduced fertility, although the available evidence remains heterogeneous [20, 48]. Long working hours have also been investigated in relation to infertility among female workers, with some evidence suggesting a higher likelihood of infertility among women with greater occupational demands [49]. These findings provide a possible context for the associations observed in the present study, although the specific occupational characteristics underlying the higher odds among employed women were not assessed.

The associations observed for education and religion were less uniform and were limited to selected categories. Religion was also associated with psychological stress and, for one category, with infertility, although these findings should be interpreted cautiously. Muslim and Sikh women showed lower odds of psychological stress compared with Hindu women, while Christian women showed lower odds of infertility. These differences should not be interpreted as evidence of a direct effect of religious affiliation on psychological or reproductive outcomes. Religious beliefs and practices may instead influence coping with stressful experiences, while family and community expectations surrounding marriage and childbearing may shape the experience of infertility and related psychological distress [50, 51]. The association with education was not consistent across categories. Compared with women with no schooling, lower odds of psychological stress were observed among women with primary education and graduation, whereas higher odds of infertility were observed among women with high school education. This variation may partly reflect differences in the characteristics and distribution of participants within the individual educational categories.

The findings suggest that infertility in this population cannot be viewed independently of psychological and social circumstances. The strong associations between stress and infertility in both models, together with the associations of RTI symptoms and physical manifestations of stress with both outcomes, indicate that women experiencing reproductive difficulties may also be dealing with considerable psychological and physical burden. The associations with financial status and employment further show that these experiences occur within a wider social and economic setting. At the same time, the inconsistent findings across sleep, education, and religion indicate that the factors associated with stress and infertility do not operate in the same way across all women. This raises an important point for interpreting infertility in this setting: focusing only on the reproductive aspect may overlook other factors that accompany or potentially interact with the experience of infertility. While the cross‐sectional design prevents determination of the direction or causality of these associations, the overall pattern observed in this study points towards a relationship between reproductive health and psychological well‐being that deserves to be considered together.

As with any research, this study has limitations that may affect the interpretation of the findings. The use of convenience sampling may limit the representativeness of the sample and restrict the generalizability of the results beyond the study population. In addition, the cross‐sectional design precludes causal inference, and the temporal relationship between stress and infertility cannot be established. The use of self‐reported measures may also have introduced recall or reporting bias, particularly in the assessment of stress and infertility history. Future studies using longitudinal designs are needed to clarify causal pathways and to determine whether stress precedes infertility or arises as a consequence of it. Further investigation into the biological mechanisms linking stress and infertility, particularly the role of stress‐induced alterations in the vaginal microbiome, may provide valuable insights for targeted preventive and therapeutic interventions. Larger, multi‐center studies and trials evaluating stress‐reduction strategies could further strengthen the clinical relevance of these findings.

## 5 Conclusion

This study highlights that infertility and psychological stress are closely connected aspects of women's reproductive health in Jammu and Kashmir. The strength and consistency of the associations observed in both directions suggest that the psychological experience of infertility deserves attention alongside the reproductive evaluation itself. At the same time, the findings indicate that infertility does not occur in isolation, but within a wider context of physical symptoms and social circumstances that may shape women's experiences. While the cross‐sectional design prevents conclusions about causality, the findings support a more integrated view of reproductive healthcare in which psychological well‐being is considered as part of, rather than separate from, infertility care. Future longitudinal research will be important to establish the temporal relationship between stress and infertility and to identify modifiable factors that could be addressed alongside conventional reproductive care.

## Author Contributions

**Kritika Sharma:** conceptualization, investigation, methodology, formal analysis, software, data curation, writing – original draft, validation. **Indu Bhushan:** conceptualization, writing – review and editing, validation, supervision, methodology. **Srinivas Sistla:** supervision, formal analysis, writing – review and editing, validation.

## Funding

The authors have nothing to report.

## Consent

Informed consent was obtained from all individual participants included in the study.

## Conflicts of Interest

The authors declare no conflicts of interest.

## Transparency Statement

The lead author, Kritika Sharma, affirms that this manuscript is an honest, accurate, and transparent account of the study being reported; that no important aspects of the study have been omitted; and that any discrepancies from the study as originally planned have been explained where applicable. Kritika Sharma accepts responsibility for the overall integrity of the work.

## Data Availability

The data that support this study cannot be publicly shared due to ethical or privacy reasons and may be shared upon reasonable request to the corresponding author if appropriate.
